# The prevalence, hospitalization outcomes and risk factors of euthyroid sick syndrome in patients with diabetic ketosis/ketoacidosis

**DOI:** 10.1186/s12902-023-01451-x

**Published:** 2023-09-12

**Authors:** Xiao-yi Deng, Min Yi, Wan-gen Li, Hui-yu Ye, Zhi-shan Chen, Xiao-dan Zhang

**Affiliations:** Department of Endocrinology, The Second Affiliated Hospital of Guangzhou Medical University, 250 East Changgang Road, Haizhu District, Guangzhou, 510260 China

**Keywords:** Euthyroid sick syndrome, Diabetic ketosis, Diabetic ketoacidosis, Hypoalbuminemia, Infection

## Abstract

**Background:**

To investigate the prevalence of euthyroid sick syndrome (ESS) and to evaluate the outcomes and risk factors associated with ESS among hospitalized patients with diabetic ketosis (DK) or diabetic ketoacidosis (DKA).

**Methods:**

Laboratory and clinical data of 396 adult hospitalized DK/DKA patients with or without ESS were collected and analyzed. Spearman linear analysis and multivariable logistic regression analyses were used to evaluate correlated factors of thyroid hormones and risk factors of ESS.

**Results:**

Most of the individuals were diagnosed with type 2 diabetes (359/396, 90.7%). The prevalence of ESS was 57.8% (229/396). Patients in ESS group were older and had a longer course of diabetes. Levels of thyroid hormones, serum lipids, and parameters reflecting acidosis were significantly decreased in ESS group. The proportion of patients with infection, acute renal injury and DKA was significantly higher in ESS group than in control group, accompanied by longer hospitalization stay and higher hospitalization costs. Free triiodothyronine positively correlates with albumin, eGFR, parameters reflecting acidosis and lipid profiles (All *P* < 0.001), and negatively correlates with age, onset age, 24-h urine albumin, hsCRP and WBC count (All *P* < 0.001). Hypoalbuminemia, low level of carbon dioxide combining power, high level of HbA1c and WBC, and co-infection are shown to be risk factors for ESS (OR = 0.866, 0.933, 1.112, 1.146, 1.929, respectively; All *P* < 0.05).

**Conclusions:**

The prevalence of ESS was high in adult DK/DKA patients. Patients with ESS had inferior clinical and socioeconomic outcomes. Early recognition and management of patients with ESS may be necessary to improve outcome.

## Background

Over the past decades, the prevalence of diabetes mellitus (DM) is increasing with the global booming of obesity and metabolic syndrome. According to recent estimates, about 536.6 million adults (10.5% of the population) suffered from diabetes globally in 2021 and the number was expected to rise to 783.2 million (12.2% of the population) in 2045 [1]. Impaired insulin secretion and varied degrees of peripheral insulin resistance is the major pathogenesis mechanism of diabetes. Insulin deficiency can increase lipolysis and along with it is the accelerated process of beta oxidation which can produce ketone bodies [2] and thus cause diabetic ketosis (DK) or diabetic ketoacidosis (DKA). Characterized as an acute complication of diabetes, DKA usually occurs in young patients with type 1 DM (T1DM) and used to be fatal in pre-insulin era. With the advancement in diagnosis and treatment, DKA is now a treatable condition. The mortality of DKA has declined dramatically these years, especially in the developed countries [3]. However, previous studies depicted that the morbidity and mortality of DKA remains high in developing countries [4]. Of note, almost one-fourth of DKA was found to be the first behaving of diabetes in older patients with type 2 DM (T2DM) [5]. A large proportion of Chinese population is still in high risk of DKA [6], which is in line with our clinical experiences. Despite of the extensively improved cure rate, DKA remains to be a significant cause of mortality in patients, especially in children and young adults. Also, DKA poses heavy burdens on health-care system. DKA is responsible for over 500,000 hospital days per year and the treatment of DKA accounts for an estimated total cost of $2.4 billion annually [2].

Euthyroid sick syndrome (ESS), also named non-thyroidal illness syndrome (NTIS) or low T3 syndrome, is characterized mainly by a reduced level of serum triiodothyronine (T3), normal or decreased level of serum thyroxine (T4) and thyroid stimulating hormone (TSH), and elevated reverse triiodothyronine (rT3) levels [7]. ESS has been reported in patients with acute and chronic illnesses such as acute myocardial infarction, trauma, postoperative and acute infection, as well as in critically ill patients [8, 9]. The scale of the decrease in serum concentration of thyroid hormones is indicated to reflect the severity of the disorder and as a result, is associated with prognosis [9]. Previously, some researchers have pointed out that the reduction of serum FT3 is positively associated with the severity of DKA in children with T1DM [10, 11]. However, fewer studies investigated the thyroid status and the associated verified outcomes in older patients and in patients with T2DM during DK/DKA, while these patients constitute the majority in our clinical practice. Therefore, we aimed to investigate the prevalence of ESS and to evaluate the outcomes and risk factors associated with ESS among adult patients with DK/DKA, not only in patients with T1DM, but also in those with T2DM.

## Methods

### Study population and design

Hospitalized patients with DK/DKA were enrolled retrospectively from the Department of Endocrinology of The Second Affiliated Hospital of Guangzhou Medical University, from January 2017 to January 2020. The study followed the Declaration of Helsinki and was approved by the Ethics Committee of The Second Affiliated Hospital of Guangzhou Medical University (Approval number 2021-hg-ks-10). Written informed consent was waived due to the retrospective nature of the study. All patients were admitted to the ward due to uncontrolled hyperglycemia complicated with DK/DKA. The diagnosis of diabetes was based on the World Health Organization (WHO) criteria [12]. DK was diagnosed in diabetic patients with blood ketone body > 3 mmol/L or positive urine ketone body, blood glucose > 11 mmol/L, and bicarbonate ion (HCO_3_^−^) ≥ 15 mmol/L or arterial power of hydrogen (pH) ≥ 7.3 [2]. The diagnosis of DKA was made if serum HCO_3_^−^ level was under 15 mmol/L and/or arterial pH level was under 7.3, and blood glucose was ranged from 16.7 to 33.3 mmol/L [2, 13]. We did not include euglycaemic ketosis or euglycaemic ketoacidosis in the study. Patients were excluded if they met one of the following exclusion criteria: (1) diagnosed with primary thyroid disease, including thyroid cancer, hyperthyroidism, and hypothyroidism; (2) severe hepatic dysfunction or chronic kidney dysfunction; (3) with history of malignant tumor; (4) with history of pituitary disease; (5) pregnancy.

### Measurement and data collection

Demographic characteristics including gender, age, onset age, duration of diabetes, previous antidiabetic medications, smoking history, and family history of diabetes were collected through the review of medical records. Body mass index (BMI) was calculated as the body weight (kg) divided by body square height (m). Blood pressure was measured in the sitting position on the right arm in line with the heart after taking a 10-min break and was recorded as an average of three times. We also collected information about the duration and cost of hospitalization of the patients. Venous blood samples were collected in the morning after an overnight fast at the second day of admission. Arterial blood sampling was performed by experienced nurses. Thyroid function tests including serum free triiodothyronine (FT3), free thyroxine (FT4), thyroid stimulating hormone (TSH), and thyroid antibodies were routinely measured, using electrochemiluminescence immunoassays. Normal ranges of thyroid hormones were as follows: TSH 0.27–4.2 mIU/L, FT3 3.10–6.80 pmol/L, and FT4 12.00–22.00 pmol/L. Patients with the serum FT3 less than 3.1 pmol/L, combined with or without a reduction of FT4 or TSH, were included in the ESS group [14, 15]. Serum analysis including fasting plasma glucose, HbA1c, fasting C-peptide, arterial gas test, blood routine test, electrolytes, hepatic and renal function, and lipid profiles were accomplished by routine laboratory methods at the Department of Clinical Laboratory. The estimated glomerular filtration rate (eGFR) was calculated according to Modification of Diet in Renal Disease equation: eGFR (mL/min/1.73 m^2^) = 186 × (SCr/88.4)^−1.154^ × (age)^−0.203^ × (0.742 if female) [16]. Urine samples of 24 h were collected to measure urine albumin levels by the biuret method. The diagnostic criteria of acute kidney injury (AKI) is a sudden increase in serum creatinine concentration by ≥ 50% within 7 days or ≥ 0.3 mg/dL (26.5 μmol/L) within 48 h, or urine volume < 0.5 ml/kg/h for > 6 h [17]. Infections (pneumonia, urinary tract infection, gastrointestinal tract infection, etc.) were diagnosed according to the clinical manifestations, laboratory and imaging examinations.

### Statistical analyses

All statistical analyses were performed using IBM SPSS software version 25.0 (IBM Corp., Armonk, New York, USA). Normally distributed data were presented as mean ± standard deviation (SD) and non-normally distributed data were presented as median (interquartile range). Categorical data were expressed as number (%). Independent sample two-tail t-test was applied for the comparisons of numeric variables with normality while the Mann–Whitney U-test was applied for the comparisons of non-normally variables. Chi-squared test was used for comparisons of categorical variables. Spearman correlation tests were conducted to evaluate associations between FT3, FT4 and other variables. Multiple logistic regression analysis was performed to identify the risk factors for ESS in DK/DKA patients. A two-tail *P* value < 0.05 was regarded as statistically significant.

## Results

### Baseline demographic and clinical characteristics of participants with and without ESS

**Fig. 1:**
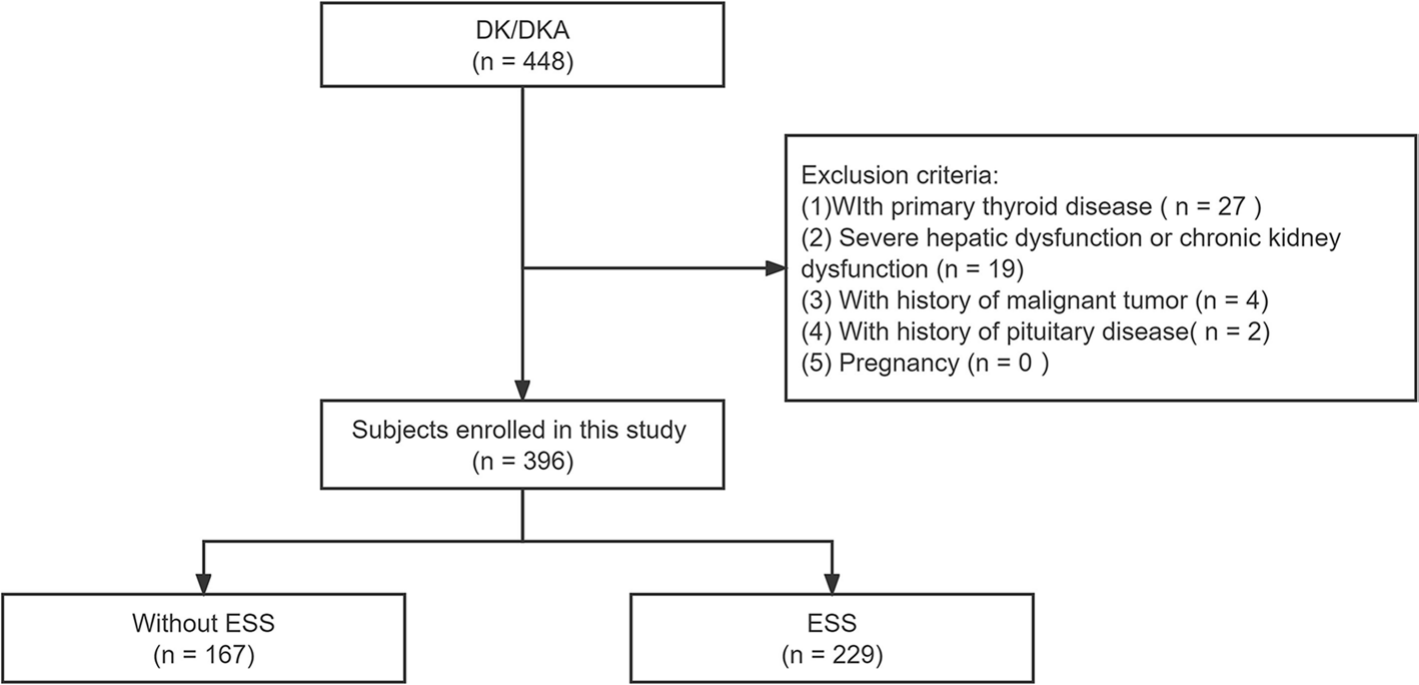
Flowchart of subject inclusion

Figure 1 shows the flowchart of subject selection. The study enrolled 396 patients with DK/DKA, including 216 males and 180 females, with a mean age of 57.5 ± 18.3 years. Table 1 displays the demographic and clinical characteristics of the patients. Patients with T2DM constituted the majority (359/396, 90.7%). No significant difference was observed in the incidence of ESS between T1DM and T2DM. Of the 396 patients, 57.8% (*n* = 229) were found to be complicated with ESS (165 with DK, 64 with DKA). Compared with subjects without ESS, individuals with ESS were older and had a longer course of diabetes. The levels of white blood cell count (WBC), neutrophil absolute value, neutrophil/lymphocyte (N/L) ratio, high sensitivity C-reactive protein (hsCRP) and creatinine were significantly higher in the ESS group than in the euthyroid group, whereas eGFR, pressure of carbon dioxide (PaCO_2_), HCO_3_^−^, carbon dioxide combining power (CO_2_CP), FT3, FT4, FT3/FT4 ratio, total cholesterol (TC), low-density lipoprotein cholesterol (LDL-C) and high-density lipoprotein cholesterol (HDL-C) levels were significantly lower in ESS group than in euthyroid group.

**Table 1:** Baseline demographic and clinical characteristics of participants with and without euthyroid sick syndrome

Variables	Overall (*n* = 396)	Without ESS (*n* = 167)	ESS (*n* = 229)	*P*-value
Gender, male/n (%)	216/396 (54.5)	100/167 (59.9)	116/229 (50.7)	0.069
Age (year)	57.5 ± 18.3	53.3 ± 17.7	60.5 ± 18.1	< 0.001
Onset age (year)	51.0 (39.0, 61.0)	49.0 (36.0, 59.0)	54 (44.0, 64.0)	0.003
Smoking history, n (%)	83 (20.5)	37 (22.3)	44 (19.2)	0.455
Family history of diabetes, n (%)	77 (19.5)	32 (19.2)	45 (19.7)	0.887
Type 1 / Type 2 DM	37/359	19/148	18/211	0.235
DK/DKA, n	302/94	137/30	165/64	0.021
Diabetic duration (year)	5.0 (0, 10.0)	2.0 (0, 10.0)	6.0 (0, 10.0)	0.004
Previous antidiabetic medications, n (%)	280 (70.7)	110 (65.9)	170 (74.2)	0.080
None	116 (29.3)	57 (34.1)	59 (25.8)	
Oral agents or GLP-1RA	129 (32.6)	43 (25.7)	86 (37.6)	
Insulin alone	84 (21.2)	37 (22.2)	47.0 (20.5)	
Both oral agents and insulin	67 (16.9)	30 (18.0)	37 (54.4)	
BMI (kg/m^2^)	23.4 ± 4.3	23.9 ± 4.0	22.9 ± 4.5	0.064
SBP (mmHg)	135.0 (119.0, 151.0)	136.0 (124.0, 151.0)	134.0 (116.0, 152.0)	0.095
DBP (mmHg)	83.8 ± 43.0	90.0 ± 64.0	76.0 ± 13.6	0.021
CO_2_CP (mmol/L)	21.9 ± 6.4	23.3 ± 4.8	20.9 ± 7.1	< 0.001
pH	7.4 ± 0.2	7.4 ± 0.1	7.4 ± 0.3	0.204
PaCO_2_ (mmHg)	33.7 ± 8.0	35.9 ± 5.7	32.1 ± 9.0	< 0.001
HCO_3_^− ^(mmol/L)	21.0 ± 5.9	22.6 ± 3.1	19.9 ± 7.0	< 0.001
K^+^ (mmol/L)	4.3 ± 6.5	3.9 ± 0.6	4.6 ± 8.5	0.291
Na^+ ^(mmol/L)	138.1 ± 61.7	141.8 ± 95.6	135.3 ± 14.1	0.303
Ca^2+^ (mmol/L)	2.2 ± 0.8	2.2 ± 0.1	2.2 ± 1.0	0.360
HbA1c (%)	12.2 (10.10, 13.80)	12.0 (10.0, 13.7)	12.4 (10.2, 13.8)	0.168
FPG (mmol/L)	11.5 ± 6.8	11.7 ± 7.1	11.3 ± 6.5	0.586
Fasting C-Peptide (μg/L)	1.6 ± 1.7	1.6 ± 1.4	1.6 ± 1.9	0.120
hsCRP (mg/L)	4.2 (1.2, 31.7)	1.6 (0.8, 4.6)	12.1 (2.1, 60.3)	< 0.001
WBC (× 10^9^/L)	8.6 (6.6, 12.5)	7.1 (6.0, 8.8)	10.7 (7.9, 15.2)	< 0.001
Neutrophil (× 10^9^/L)	6.2 (4.2, 10.3)	4.4 (3.4, 6.2)	8.15 (5.3, 12.2)	< 0.001
Lymphocyte (× 10^9^/L)	1.6 (1.1, 2.1)	1.72 (1.30, 2.20)	1.48 (0.99, 1.97)	< 0.001
N/L ratio	4.0 (2.3, 7.7)	2.7 (1.79, 4.04)	5.6 (3.47, 10.13)	< 0.001
Albumin (g/L)	35.3 (31.6, 38.4)	37.6 (35.1,40.0)	33.5 (28.9, 36.2)	< 0.001
AST (IU/L)	28 ± 63	29 ± 68	28 ± 58	0.878
ALT (IU/L)	29 ± 59	33 ± 70	25 ± 49	0.195
Triglyceride (mmol/L)	2.3 ± 3.6	2.7 ± 4.4	2.0 ± 2.7	0.056
Total cholesterol (mmol/L)	4.7 ± 1.7	5.0 ± 1.6	4.5 ± 1.7	0.008
LDL-C (mmol/L)	3.0 ± 1.2	3.2 ± 1.3	2.9 ± 1.2	0.030
HDL-C (mmol/L)	1.0 ± 0.4	1.1 ± 0.3	1.0 ± 0.4	0.027
Uric acid (μmol/L)	350 ± 140	337 ± 111	340 ± 158	0.115
Creatinine (μmol/L)	78.2 (69.9, 106.0)	75.1 (59.0, 96.0)	81.4 (62.3, 117.0)	0.039
eGFR (mL/min/1.73 m^2^)	83.3 (58.9, 104.9)	89.1 (64.3, 109.2)	79.3 (55.4,102.6)	0.001
24-h urine albumin (mg)	56.9 ± 107.5	45.47 ± 116.3	67.6 ± 101.8	0.083
FT3 (pmol/L)	2.9 ± 0.9	3.8 ± 0.6	2.3 ± 0.5	< 0.001
FT4 (pmol/L)	14.8 ± 6.0	16.1 ± 3.3	13.9 ± 7.2	< 0.001
FT3/FT4 ratio	0.20 (0.16, 0.24)	0.24 (0.20, 0.26)	0.17 (0.15, 0.20)	< 0.001
TSH (μIU/mL)	1.5 ± 2.0	1.4 ± 0.8	1.6 ± 2.6	0.423
TgAb (IU/mL)	10.0 (1.24, 10.00)	10.0 (1.17, 10.00)	8.6 (1.27, 10)	0.714
TPOAb (IU/mL)	5.8 (0.25, 14.31)	8.0 (0.25, 13.89)	2.7 (0.24, 14.62)	0.892
Positive thyroid autoantibodies, n (%)	32 (9.6)	7 (5.3)	25 (12.4)	0.032

Continuous variables with normal distribution were presented as mean ± standard deviation and non-normal distributed variables were presented as median (interquartile range). Categorical variables were presented as n (%). Independent sample two-tail t-test was applied for the comparisons of numeric variables with normality while Mann–Whitney U-test was applied for the comparisons of non-normally variables. Chi-squared test was used for comparisons of categorical variables

*n* number, *DM* diabetes mellitus, *DK* diabetic ketosis, *DKA* diabetic ketoacidosis, *GLP-1RA* glucagon-like peptide-1 receptor agonist, *BMI* body mass index, *SBP* systolic blood pressure, *DBP* diastolic blood pressure, *CO2CP* carbon dioxide combining power, *pH* power of hydrogen, *PaCO2* pressure of carbon dioxide, *HCO3-* bicarbonate ion, *HbA1c* glycated hemoglobin, *FPG* fasting plasma glucose, *hs-CRP* high sensitivity C-reactive protein, *WBC* White blood cell count, *N/L* ratio Neutrophil / Lymphocyte ratio, *AST* aspartate aminotransferase, *ALT* alanine transaminase, *LDL-C* low-density lipoprotein cholesterol, *HDL-C* high-density lipoprotein cholesterol, *eGFR* estimated glomerular filtration rate, *FT3* free triiodothyronine, *FT4* free thyroxine, *TSH* thyroid stimulating hormone, *TgAb* anti-thyroglobulin antibodies, *TPOAb* thyroid peroxidase antibody

### Clinical and socioeconomic outcomes of DK/DKA patients in ESS group and without ESS group

Considering the possible negative impact of ESS, we compared clinical and socioeconomic outcomes in the two groups. As shown in Table 2, the duration of hospital stay (9 (7, 11) day vs. 8 (7, 10) day, *P* = 0.018) and hospitalization costs (11,833 (9598, 15,974) yuan vs. 10,272 (8456, 11,718) yuan, *P* < 0.001) were significantly higher in individuals with ESS than in euthyroid individuals. Importantly, the occurrence of comorbidities including co-infection, the prevalence of AKI and DKA was also significantly higher in the ESS group (*P* < 0.001, *P* = 0.009, *P* = 0.021, respectively).

**Table 2:** Clinical features of DK/DKA patients in different groups

Variables	Without ESS	ESS	*P*
Hospitalization costs (yuan)	10,272 (8456, 11,718)	11,833 (9598, 15,974)	< 0.001
Hospital stay (day)	8 (7, 10)	9 (7, 11)	0.018
Co-infection, n (%)	45 (27.4)	150 (67.0)	< 0.001
Acute kidney injury, n (%)	19 (11.6)	49 (21.7)	0.009
Diabetic ketoacidosis, n (%)	30 (18.0)	64 (27.9)	0.021

Hospitalization costs and hospital stay were expressed as median (interquartile range). Acute kidney injury and diabetic ketoacidosis were presented as n (%). Mann–Whitney U-test was applied for the comparisons of non-normally variables. Chi-squared test was used for comparisons of categorical variables

### Analysis of correlated factors of thyroid hormones

**Fig. 2:**
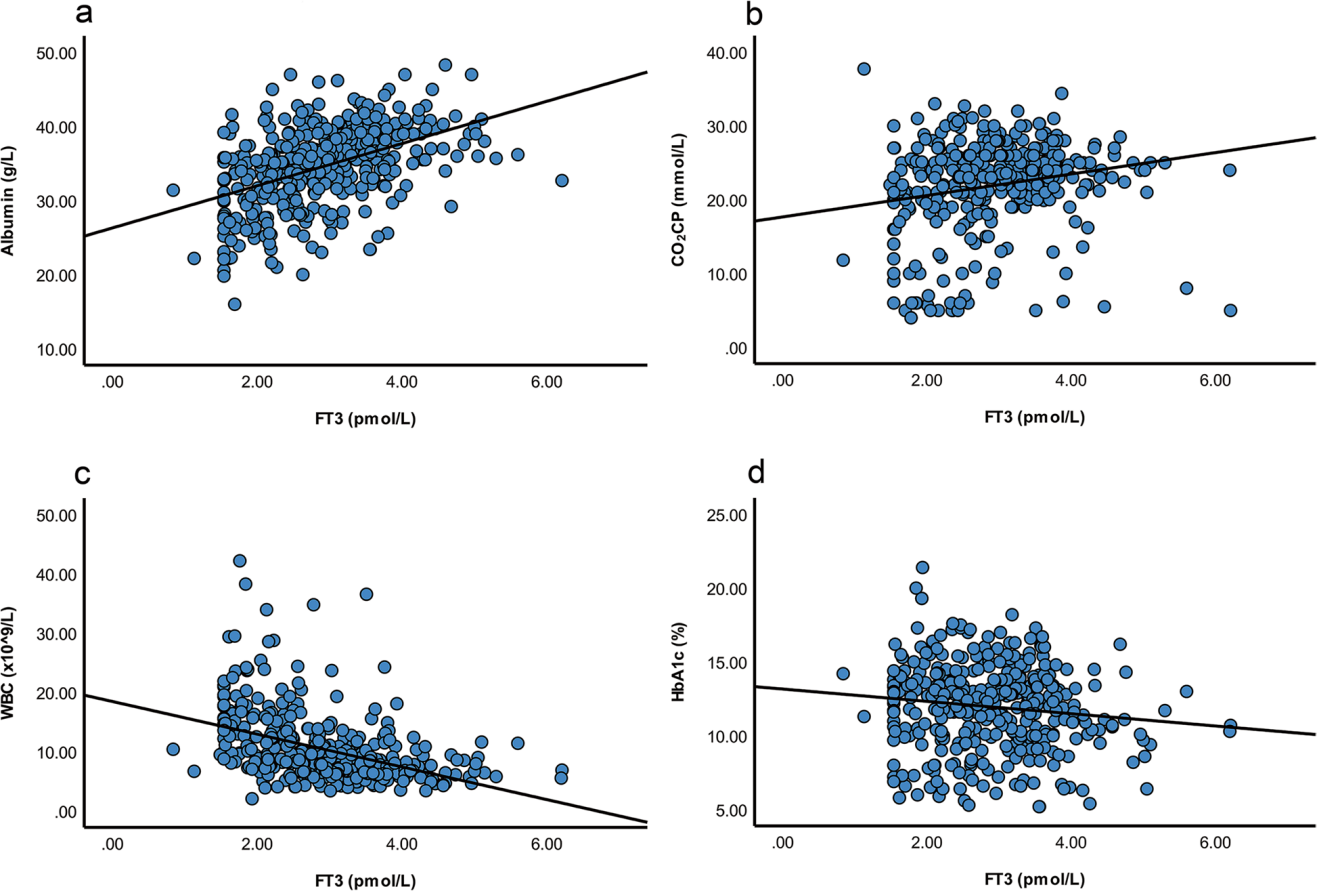
Correlations between FT3 and albumin (**a**), CO_2_CP (**b**), WBC (**c**), HbA1c (**d**). Within patients, there were significantly correlations between FT3 and albumin, CO_2_CP, WBC, HbA1c, respectively (*r* = 0.477, 0.235, -0.504, -0.117, *P* < 0.05). Abbreviations: HbA1c, glycated hemoglobin; WBC, white blood cell count; FT3, free triiodothyronine

The relationships between FT3, FT4 levels and clinical factors were presented in Table 3. Both FT3 and FT4 positively correlated with levels of BMI, serum albumin, pH, PaCO_2_, CO_2_CP, and HCO_3_^−^, and negatively correlated with WBC, neutrophils, and N/L ratio (All *P* < 0.05). FT3 was also positively related to eGFR, lymphocyte, TC, LDL-C, and HDL-C, and was negatively related to age, onset age, diabetes duration, HbA1c, creatinine, 24-h urine albumin, and hsCRP (All *P* < 0.05). But FT4 correlated with none of these parameters. The associations between FT3 and represented factors including CO_2_CP, albumin, WBC, and HbA1c were shown in Fig. 2 (*r* = 0.236, 0.480, -0.502, -0.119, respectively; All *P* < 0.05).

**Table 3:** Correlating factors of FT3 and FT4 in DK/DKA patients

Variables	FT3		FT4	
	Correlation coefficient	*P*	Correlation coefficient	*P*
Age	-0.201	< 0.001	-0.051	0.311
Onset age	-0.176	< 0.001	-0.049	0.332
Diabetes duration	-0.107	0.036	0.000	0.998
BMI	0.155	0.016	0.149	0.020
HbA1c	-0.117	0.024	-0.008	0.881
Albumin	0.477	< 0.001	0.188	< 0.001
Creatinine	-0.108	0.033	0.003	0.948
eGFR	0.180	< 0.001	0.025	0.627
24-h urine albumin	-0.215	< 0.001	-0.053	0.367
pH	0.172	0.004	0.147	0.015
PaCO_2_	0.309	< 0.001	0.166	0.009
CO_2_CP	0.235	< 0.001	0.170	0.001
HCO_3_^−^	0.285	< 0.001	0.178	0.004
hsCRP	-0.440	< 0.001	-0.054	0.406
WBC	-0.504	< 0.001	-0.200	< 0.001
Neutrophil	-0.542	< 0.001	-0.215	< 0.001
Lymphocyte	0.234	< 0.001	0.094	0.063
N/L ratio	-0.522	< 0.001	-0.226	< 0.001
Triglyceride	0.045	0.372	-0.031	0.542
Total cholesterol	0.197	< 0.001	0.049	0.334
LDL-C	0.155	0.002	0.030	0.556
HDL-C	0.232	< 0.001	0.155	0.002

Spearman correlation tests was applied for evaluating associations between FT3, FT4 and other variables

*FT3* Free triiodothyronine, *FT4* Free thyroxine, *BMI* Body mass index, *HbA1c* Glycated hemoglobin, *pH* Power of hydrogen, *PaCO*_*2*_ Pressure of carbon dioxide, *CO*_*2*_*CP* Carbon dioxide combining power, *HCO*_*3*_^*−*^ Bicarbonate ion, *hs-CRP* High sensitivity C-reactive protein, *WBC* White blood cell count, *N/L* *ratio* Neutrophil/Lymphocyte ratio, *LDL-C* Low-density lipoprotein cholesterol, *HDL-C* High-density lipoprotein cholesterol

### Risk factors for ESS patients with DK/DKA

After adjusting various confounding factors, multiple logistic regression presented associated factors of ESS including HbA1c (OR = 1.112, *P* = 0.040, 95%CI 1.005 ~ 1.231), CO_2_CP (OR = 0.933, *P* = 0.007, 95%CI 0.888 ~ 0.981), albumin (OR = 0.866, *P* < 0.001, 95%CI 0.814 ~ 0.921), WBC (OR = 1.146, *P* = 0.002, 95%CI 1.052 ~ 1.248), and co-infection (OR = 1.929, *P* = 0.031, 95%CI 1.063 ~ 3.500) (Table 4). Patients with severe acidosis, hypoalbuminemia, high WBC count and co-infection were more likely to present ESS.

**Table 4:** Risk factors of euthyroid sick syndrome in DK/DKA patients

Variables	B	S.E	Wald	*P*	OR	95%CI for EXP(B)	
						Lower	Upper
Age	0.014	0.009	1.911	0.074	1.014	0.994	1.033
HbA1c	0.106	0.052	4.236	0.040	1.112	1.005	1.231
eGFR	-0.003	0.004	0.534	0.465	0.997	0.989	1.005
CO_2_CP	-0.069	0.025	7.406	0.007	0.933	0.888	0.981
Albumin	-0.144	0.032	20.659	< 0.001	0.866	0.814	0.921
WBC	0.136	0.044	9.691	0.002	1.146	1.052	1.248
Co-infection	0.657	0.304	4.666	0.031	1.929	1.063	3.500
HDL-C	0.039	0.411	0.009	0.924	1.040	0.465	2.326

The risk factors for ESS in DK/DKA patients were analyzed using multiple logistic regression estimation

*HbA1c* Glycated hemoglobin, *CO*_*2*_*CP* Carbon dioxide combining power, *WBC* White blood cell count

## Discussion

As expected, the present study revealed a high prevalence of ESS in hospitalized patients with DK/DKA. Different from previous studies which mainly focused on young patients with T1DM [10, 11, 18], most of the patients were middle-aged and elderly patients with T2DM in this research. Moreover, patients with ESS were older in age and had a longer course of diabetes. But the prevalence of ESS in our study was comparable to the ones in previous reports.

ESS is considered to be an independent risk factor for the severity of illness and its prognosis [9, 19–21]. Previous studies have shown that the reduction of FT3 and FT4 predicted the severity of illness and hospital mortality rates of patients [22]. The changes of thyroid hormone levels in ESS are not associated with primary thyroid disease [19–21, 23]. The reduction of FT3 is an adaptive response to stress during the acute phase response in critical illness [24, 25] and the serum level of FT3 could return to normal with treatment and recovery of illness [26]. Shao et al. found that the ESS patients with DKA in children had lower serum FT3, FT4 and TSH, accompanied with worse glucose control [11]. Previous studies demonstrated high prevalence of thyroid dysfunction in DKA patients [22, 27]. These findings were in accordance with our study. In the present research, patients with ESS had lower levels of serum FT3 and FT4, and higher levels of HbA1c. As for the hospitalized outcomes, all the patients got recovered from DK/DKA and thus the mortality rate was 0% in the study. However, much heavier socioeconomic burdens were indicated in the ESS group by a longer hospital stay and higher hospitalization costs. Also, the occurrence of comorbidities including AKI and co-infection were significantly higher in patients with ESS.

The changes in thyroid function can affect renal function directly, as well as make indirect alterations by affecting systemic hemodynamics, metabolism, and cardiac function [28, 29]. The relationships between thyroid hormones and renal function have been broadly recognized these years. But most of the evidences came from patients with chronic renal impairment [28–31]. Previously, a study reported a high prevalence of ESS (about 70%) in patients with AKI [29]. As known, DK/DKA is a direct imposing factor for AKI. Other studies also documented the high prevalence of AKI in DK/DKA [32, 33]. But few researches explored the the correlation between ESS and AKI in DK/DKA. In our study, we found a higher occurrence of AKI in patients with ESS compared with euthyroid patients. Serum creatinine, eGFR and 24-h urine albumin was significantly associated with serum FT3 level. However, due to the complicated systemic alterations (hemodynamics, metabolism, etc.), the cause-and-effect relationship between changes of thyroid hormones and kidney function in acute diseases has not been well-established based on current evidences. Further studies are needed to elucidate the associations.

In the present study, we assessed parameters reflecting the extent of acidosis, including pH, PaCO_2_, HCO_3_^−^ and CO_2_CP, to evaluate the association between acidosis and the levels of thyroid hormones. We noticed that DK/DKA patients with ESS have higher levels of acidosis. The values of pH and CO_2_CP were positively associated with both FT3 and FT4 levels. And the prevalence of ESS increased with higher degree of acidosis. Previous studies described similar changes of thyroid hormones in DKA patients [10, 26]. Rashidi et al. pointed out that the lower level of pH in DKA patients, the lower level FT3 [26]. The metabolic acidosis may play important role in the formation of ESS by affecting the thyroid hormone metabolism [34]. Also, CO_2_CP was manifested as a significant independent risk factor for ESS in DK/DKA patients in our study. Together, these results indicated a connection between acidosis state and the alterations in thyroid hormones in DK/DKA patients.

Serum albumin was suggested to strongly positively correlate with thyroid hormone (FT3 and FT4) levels in DK/DKA patients in the present study. Albumin is known as a major plasma protein. T3 levels decline in the early stage of ESS, and the decrease of T4 to T3 conversion is related to the reduction in albumin levels [35]. Low albumin level is widely used as a predictor of malnutrition. According to previous researches, malnutrition may be one of the factors affecting thyroid hormones [36, 37]. But in our study, most of the patients were in fine nutritional status. In fact, serum albumin levels may also be influenced by various factors such as liver diseases and changes of intravascular volume [38]. They may decrease as a result of inflammation due to acute or chronic diseases [39]. Therefore, serum albumin has been validated as a measurement of disease severity in the Acute Physiology and Chronic Health Evaluation scoring system [40]. The degradation of albumin level was shown to be an independent predictor of ICU mortality in previous studies [37]. Hypoalbuminemia was also found to be associated with ESS in various kinds of diseases (rheumatoid arthritis [41], COVID-19 infection [42], acute pancreatitis [43], etc.). Serum albumin levels were shown to be reduced in ESS pediatric patients with DK/DKA [27]. Besides the aforementioned influencing factors, the deficiency of insulin can also cause a drop in albumin in DK/DKA, since albumin synthesis in hepatocytes depends on sufficient insulin secretion [11, 44]. In the present research, albumin deficiency was also indicated to be an independent risk factor for ESS, and its reduction might present a worse prognosis in DK/DKA patients.

Inflammation is considered as one of the precipitating factors for many pathological circumstances including DK/DKA [45, 46]. It has been reported that DKA correlated with active systemic inflammatory response and oxidative stress [47, 48]. Elevations in nonspecific inflammatory cytokines were found to correlate strongly and positively with DK/DKA [49–51]. Previous studies also pointed out that serum levels of thyroid hormones were negatively associated with the serum concentrations of inflammatory cytokines [52]. In the present study, inflammatory indicators, including WBC, neutrophils, hsCRP and N/L ratio, were significantly higher in individuals with ESS, and were negatively associated with FT3. Moreover, elevated WBC and co-infection were risk factors for ESS in DK/DKA. Previous studies have also proposed other factors associated with ESS, including uric acid, serum lipids and other metabolic indicators [53–55]. But in this study, we did not have similar findings.

This study has several limitations. First, it was single-center research with a relatively small number of patients. Second, the cause-and-effect relationship could not be built due to the retrospective nature of the study. Third, follow-up was not done to evaluate the dynamic change of thyroid hormones in ESS patients after recovering of DK/DKA. Therefore, the results need to be cautiously interpreted.

## Conclusion

This study demonstrated a high prevalence of ESS in adult DK/DKA patients with elder ages, most of whom were with T2DM. Patients with ESS had inferior clinical and socioeconomic outcomes. Low albumin levels, high WBC counts, poor glycemic control, co-infection, and higher levels of acidosis were risk factors of ESS in DK/DKA patients. Diabetic education, early detection and treatment of DK/DKA is necessary not only for patients with T1DM, but also for patients with T2DM. Early interventions for patients with the identified risk factors for ESS might be helpful to improve hospitalization outcomes.

## Data Availability

The data used for the current study are available from the corresponding author on reasonable request.

## Abbreviations

ESS: Euthyroid sick syndrome
DK: Diabetic ketosis
DKA: Diabetic ketoacidosis
DM: Diabetes mellitus
T1DM: Type 1 DM
T2DM: Type 2 DM
NTIS: Non-thyroidal illness syndrome
T3: Triiodothyronine
T4: Thyroxine
TSH: Thyroid stimulating hormone
FT3: Free triiodothyronine
FT4: Free thyroxine
rT3: Reverse triiodothyronine
WHO: World Health Organization
HCO3^−^: Bicarbonate ion
pH: Power of hydrogen
BMI: Body mass index
eGFR: Estimated glomerular filtration rate
AKI: Acute kidney injury
SD: Standard deviation
WBC: White blood cell count
N/L ratio: Neutrophil/lymphocyte ratio
hsCRP: High sensitivity C-reactive protein
PaCO2: Pressure of carbon dioxide
CO2CP: Carbon dioxide combining power
TC: Total cholesterol
LDL-C: Low-density lipoprotein cholesterol
HDL-C: High-density lipoprotein cholesterol
